# The expression regulation of *recA* gene and bacterial class 2 integron-associated genes induced by antibiotics

**DOI:** 10.3389/fmicb.2025.1632813

**Published:** 2025-08-29

**Authors:** Jinglu Ye, Qian Sun, Qiaoping Wu, Jianqiang Xu, Ye Yang, Rongqing Zhao, Qingcao Li

**Affiliations:** ^1^Department of Clinical Laboratory, The Affiliated LiHuiLi Hospital of Ningbo University, Ningbo, China; ^2^State Key Laboratory for Diagnosis and Treatment of Severe Zoonostic Infectious Disease, Wuhan, China

**Keywords:** class 2 integron, *recA*, resistance genes, integrase gene, sub-MIC, antibiotic

## Abstract

**Objective:**

To investigate the effects and mechanisms of common antibiotics induction on the expression of class 2 integron integrase and variable region resistance genes in bacteria, as well as potential structural mutations.

**Methods:**

Clinical isolates containing non-functional class 2 integrons and functional class 2 integrons were selected. Strains containing non-functional class 2 integrons or functional class 2 integrons were constructed using isolated DNA templates. These strains were subjected to continuous induction with drug concentrations of 1/2 MIC and 1/4 MIC (ciprofloxacin, ampicillin, and kanamycin) and a concentration of 0.2 μg/ml (mitomycin C) over 8 days. The relative expression levels of relevant genes were measured on days 1, 3, and 8. Drug resistance in the experimental strains was assessed before and after induction to identify any differences. Finally, the sequence of the non-functional class 2 integron integrase gene was analyzed for structural changes that occurred as a result of induction.

**Results:**

All drugs selected in this study increased the relative expression levels of *recA, intI2, dfrA1, sat2*, and *aadA1*. Significant differences in inductive abilities were observed among the drugs. The 1/2 MIC concentrations were more effective than 1/4 MIC concentrations in increasing the relative expression levels of target genes and enhancing the resistance of the experimental strains. The relative expression levels of *recA, intI2*, and *dfrA1* rose on day 1, peaked on day 3, and slightly declined by day 8. Induced strains exhibited increased resistance to the drugs, with the most significant changes observed in the clinical isolates, particularly concerning CIP resistance. Notably, clinical isolate 7b induced with 1/2 MIC KAN exhibited the loss of one base at position 12bp in the integrase sequence. However, none of the four drugs induced mutations at the 444 bp position of class 2 integrons.

**Conclusion:**

Sub-MIC concentrations of drugs have been shown to induce an increase in the relative expression level of the SOS response-related gene *recA*, as well as the integrase and resistance genes of class 2 integrons. Continuous induction leads to sustained upregulation of these genes, which stabilizes or slightly decreases upon reaching a plateau. However, the capacity of different drugs to induce expression varies significantly. Short-term antibiotic exposure did not result in critical mutations that convert class 2 integrons into functional forms.

## 1 Introduction

Integrons (Stokes and Hall, 1989; Akrami et al., 2019; Ghaly et al., 2022) are natural cloning and expression vectors that are common multifunctional gene capture systems in bacterial genomes that enable bacteria to generate phenotypic diversity and form adaptive responses (Partridge et al., 2018; Ahmadian et al., 2020; Ali et al., 2020; Bhat et al., 2023). This capability plays a vital role in the emergence and spread of antibiotic resistance. Class 1 integrons are most commonly seen in clinical settings, followed by class 2 integrons (Jové et al., 2017; Ahmadian et al., 2020). The majority of class 2 integrons currently isolated from clinical samples are non-functional (Jiang et al., 2023), their integrases cannot translate the complete integrase protein, rendering them unable to integrate and excise antibiotic resistance gene cassettes. Functional class 2 integrons (Márquez et al., 2008; Wei et al., 2014; Lu et al., 2022) are a unique subset characterized by a mutation in the integrase gene that replaces a stop codon (TAA), inserted after the 178th amino acid, with a codon for an active amino acid. This mutation enables the translation and synthesis of a complete integrase protein, facilitating the integration and excision of resistance gene cassettes. Functional class 2 integrons are extremely rare in clinical environments (Lu et al., 2022). Gene cassettes carried by integrons have evolved antimicrobial resistance in key clinical environments (Bhat et al., 2023). Class 2 integrons typically harbor three resistance gene cassettes: *dfrA1, sat2*, and *aadA1* (Jové et al., 2017). The *dfrA1* gene encodes dihydrofolate reductase (DHFR), which mediates resistance to trimethoprim. The *sat2* gene encodes streptothricin acetyltransferase, which confers resistance by transferring an adenylyl group to the streptothricin molecule, thereby inactivating it. The *aadA1* gene encodes aminoglycoside adenylyltransferase, which modifies specific sites on aminoglycoside antibiotics (such as streptomycin and spectinomycin), preventing their binding to bacterial ribosomes.

Studies on class 1 integrons have revealed that various drugs can trigger the SOS response, which collaborates with host regulatory networks to control the expression of integrases and resistance gene cassettes. This mechanism ultimately transforms integrons into a genetically stable and efficient system, endowing bacteria with a low-cost, highly adaptable evolutionary potential (Ahmadian et al., 2020; Mohanraj and Mandal, 2022). However, current research on class 2 integrons mainly focuses on their clinical distribution and the types and structures of gene cassettes in the variable region (Jové et al., 2017; Alonso et al., 2018). Reports on functional class 2 integrons are rare. The mechanisms underlying the TAA to CAA conversion in the integrase gene, the expression status and regulatory mechanisms of functional class 2 integrase, and its relationship with variable region gene cassettes remain poorly understood. Therefore, this study aims to investigate the mutation and expression regulation of class 2 integron integrase genes under the induction of common drugs. Furthermore, the research explores the role of these drugs in modulating the mutation and expression of both class 2 integron and functional class 2 integron integrase genes and examines their correlation with gene cassettes in the variable region.

## 2 Materials and methods

### 2.1 Selection of experimental strains and plasmids construction

In this study, one strain of non-functional class 2 integron-positive and functional class 2 integron-positive bacteria was selected, both clinically isolated and designated as 7b and 5b, respectively. Both strains are *Proteus mirabilis* and are class 1 integron-negative (Our research group had previously successfully identified 3 functional class 2 integron-positive strains and 61 non-functional class 2 integron-positive strains from clinically isolated *Proteus mirabilis*). This study was conducted following the principles of the Helsinki Declaration and obtained approval from the Ethics Committee of Li Huili Hospital, Ningbo City (Approval No. KY2024SL306-01). Clinical DNA from the integron-positive strains was used as a template to construct recombinant plasmids, which were subsequently transformed into *Escherichia coli* TOP10. The bacteria containing the recombinant plasmids were named pAC-FA1, pAC-FA3, and pAC-FA6. The strains used in this study are detailed in Table 1.

**Table 1:** Strains used in this study.

**Strains**	**Genotype or description**	**Source**
5b	*intI2* (functional) - *dfrA1*- *sat2*- *aadA1*- *orfX*	Laboratory collection
7b	*intI2* (non-functional) - *dfrA1*- *sat2*- *aadA1*- *orfX*	Laboratory collection
pAC-FA1	*intI2* (non-functional) - *dfrA1*	This study
pAC-FA3	*intI2* (functional) - *dfrA1*	This study
pAC-FA6	*intI2* (functional) - *dfrA1*- *sat2*- *aadA1*	This study
*E. Coli* TOP10	F- *mcrA Δ(mrr-hsdRMS-mcrBC) ϕ80 lacZΔM15Δ lacX74 recA1 araΔ139Δ(ara-leu)7697 galU galK rpsL (StrR)endA1 nupG*	purchased from Shanghai Sangon Biotechnology Co., Ltd.

The construction of the strains was carried out as follows: The integron genes from clinically isolated strains 7b and 5b were used as DNA templates for the design of corresponding primers to amplify and purify target fragments. Plasmid pACYC184 was extracted and double-digested using the restriction enzymes *Ase I* and *Hind III*. The enzyme-digested products were then verified and purified through electrophoresis. According to the seamless cloning reaction system, the target gene fragments were ligated with the enzyme-digested plasmids to form recombinant plasmids, which were subsequently transformed into *E. coli* TOP10 competent cells. Post-transformation, the bacteria were spread onto LB plates containing chloramphenicol (25 μg/ml) and cultured overnight. Successful transformation of the plasmid into *E. coli* TOP10 was confirmed by the growth of the bacteria on chloramphenicol plates, as plasmid pACYC184 contains the chloramphenicol resistance gene (*cat*). Finally, plasmids were extracted from selected colonies for sequencing validation. The oligonucleotide primers used in this study are listed in Supplementary Table S1. Restriction enzymes *Ase I* and *Hind III* were purchased from SibEnzyme Ltd. Plasmid extraction kits and competent cells were purchased from Shanghai Sangon Biotechnology Co., Ltd.

### 2.2 Antibiotic sensitivity testing of experimental strains

In this study, four drugs were selected: ciprofloxacin (CIP), ampicillin (AMP), kanamycin (KAN), and mitomycin C (MMC). The minimum inhibitory concentrations (MICs) of CIP, AMP, and KAN were determined using the broth microdilution method, following the guidelines of the Clinical and Laboratory Standards Institute (CLSI) (CLSI, 2022), for subsequent antibiotic induction experiments. Antibiotic susceptibility of the experimental strains was assessed before and after drug induction using the Kirby-Bauer disk diffusion method (KB method) to compare changes in resistance. During the experiment, *E. coli* ATCC25922 was utilized as the quality control strain, and *E. coli* TOP10 served as the negative control strain. Antibiotic susceptibility discs were purchased from Oxoid Limited, and the antibiotics were purchased from Shanghai Sangon Biotechnology Co., Ltd.

### 2.3 Experimental strains for drug induction

The experimental strains were induced for 8 consecutive days to explore the changes in the relative expression of the target genes and antibiotic resistance. For CIP, AMP, and KAN, two concentrations, 1/2 MIC and 1/4 MIC, were utilized. The induction concentration for MMC was set at 0.2 μg/ml (Baharoglu et al., 2012). The induction process involved mixing LB liquid medium containing the specified drug concentrations with the experimental strains, followed by overnight incubation at 37 °C with shaking. The following day, a fresh LB liquid medium containing the same antibiotic concentration (5 ml) was prepared, and 50 μl of the overnight cultured bacteria were added and incubated overnight. This procedure was repeated daily for a total of 8 days of continuous culture.

### 2.4 Quantitative RT-PCR (RT-qPCR) detection of relative gene expression

The study aimed to explore the relative transcription levels of *recA, intI2*, and variable region resistance genes (*dfrA1, sat2*, and *aadA1*) in the experimental strains following drug induction. Bacterial RNA was extracted using a column-based total RNA extraction and purification kit from 1 ml of overnight bacterial culture on days 1, 3, and 8 of induction. The extracted RNA was purified and subjected to reverse transcription (RT) to obtain cDNA templates. RT-qPCR was conducted to monitor the expression levels of selected genes throughout the induction process using an ABI7500 real-time quantitative PCR instrument. A blank control was included on each plate, with double-distilled H_2_O replacing the template cDNA. The internal reference gene of clinically isolated strains was *16S rRNA*, while for constructed strains, it was *cat*. All experiments were performed in triplicate (*n* = 3 biological replicates) to ensure reproducibility. Relative expression levels were calculated using the 2^−Δ*ΔCt*^ method and each reaction was performed in triplicate. The oligonucleotide primers used in this study are listed in Supplementary Table S1. The reagents used in the process were purchased from Shanghai Sangon Biotechnology Co., Ltd.

### 2.5 Analysis of structural changes in class 2 integron integrase

The constructed strains pAC-FA1 and clinically isolate 7b were selected for analysis both before and after induction. Primers were designed to amplify the class 2 integron integrase, and the resulting purified product was sent to Shanghai Sangon Biotech Company for sequencing. The sequencing results were analyzed using Vector NTI software to compare the integrase gene sequences of the integrons before and after induction. Additionally, potential amino acid sequences were inferred based on these gene sequences, in conjunction with changes in transcription and expression levels of the integrase and variable region genes observed before and after induction.

### 2.6 Statistical analysis

Data were tabulated and analyzed using Origin 2021. Statistical analysis between the experimental and control groups was performed using one-way ANOVA, followed by Tukey's multiple comparison test. A *P*-value of < 0.05 was considered statistically significant.

## 3 Results

### 3.1 Original MIC of experimental strains

Prior to drug induction, the MIC of the experimental strains was determined using the broth microdilution method, with ciprofloxacin (CIP), ampicillin (AMP), and kanamycin (KAN) being tested (Table 2). The constructed strains showed sensitivity to all three drugs. Clinical isolates were intermediate in response to KAN and susceptible to CIP and AMP (Supplementary Table S2).

**Table 2:** The original MIC of the experimental strains.

**Experimental strain**	**CIP (μg/ml)**	**AMP (μg/ml)**	**KAN (μg/ml)**
pAC-FA1	0.008	2	32
pAC-FA3	0.008	2	32
pAC-FA6	0.008	2	32
5b	0.512	2	64
7b	1.024	2	128

CIP, ciprofloxacin; AMP, ampicillin; KAN, kanamycin.

### 3.2 Relative expression and trends of *recA* after induction

**Figure 1:**
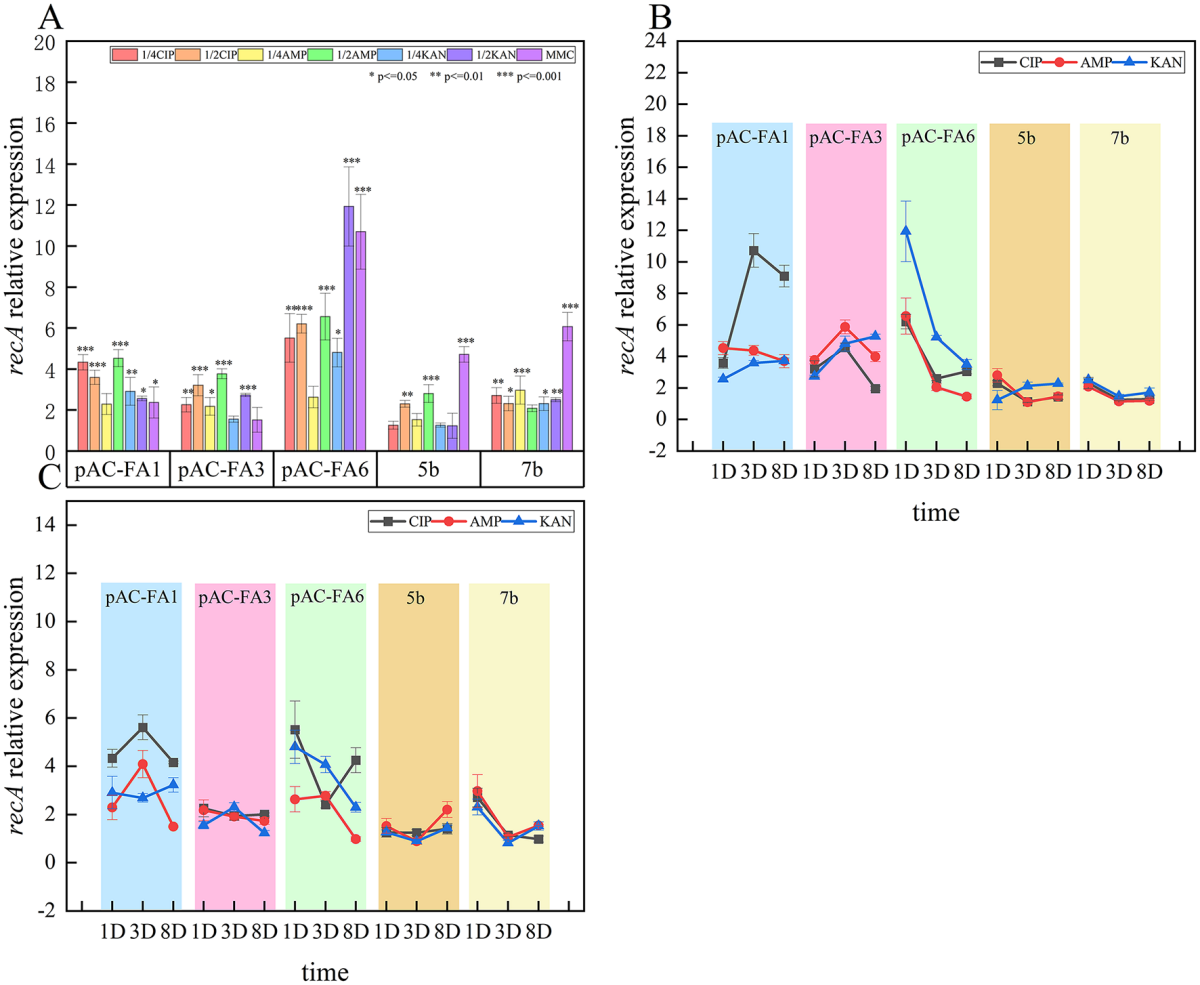
Relative expression and trends of *recA*. **(A)** Relative expression level of *recA* in experimental strains 1 day post-induction with different drugs **(B)** Changes in relative expression level of *recA* in experimental strains on the 1st, 3rd, and 8th days at a 1/2 MIC concentration **(C)** Changes in relative expression level of *recA* in experimental strains on the 1st, 3rd, and 8th days at a 1/4 MIC concentration. The vertical axis values represent the ratio of relative expression of *recA* in the experimental group to the control group. ***P < 0.001, **P < 0.01, *P < 0.05. The *p*-values and effect sizes for the line graph are listed in Supplementary Table S3.

**Figure 2:**
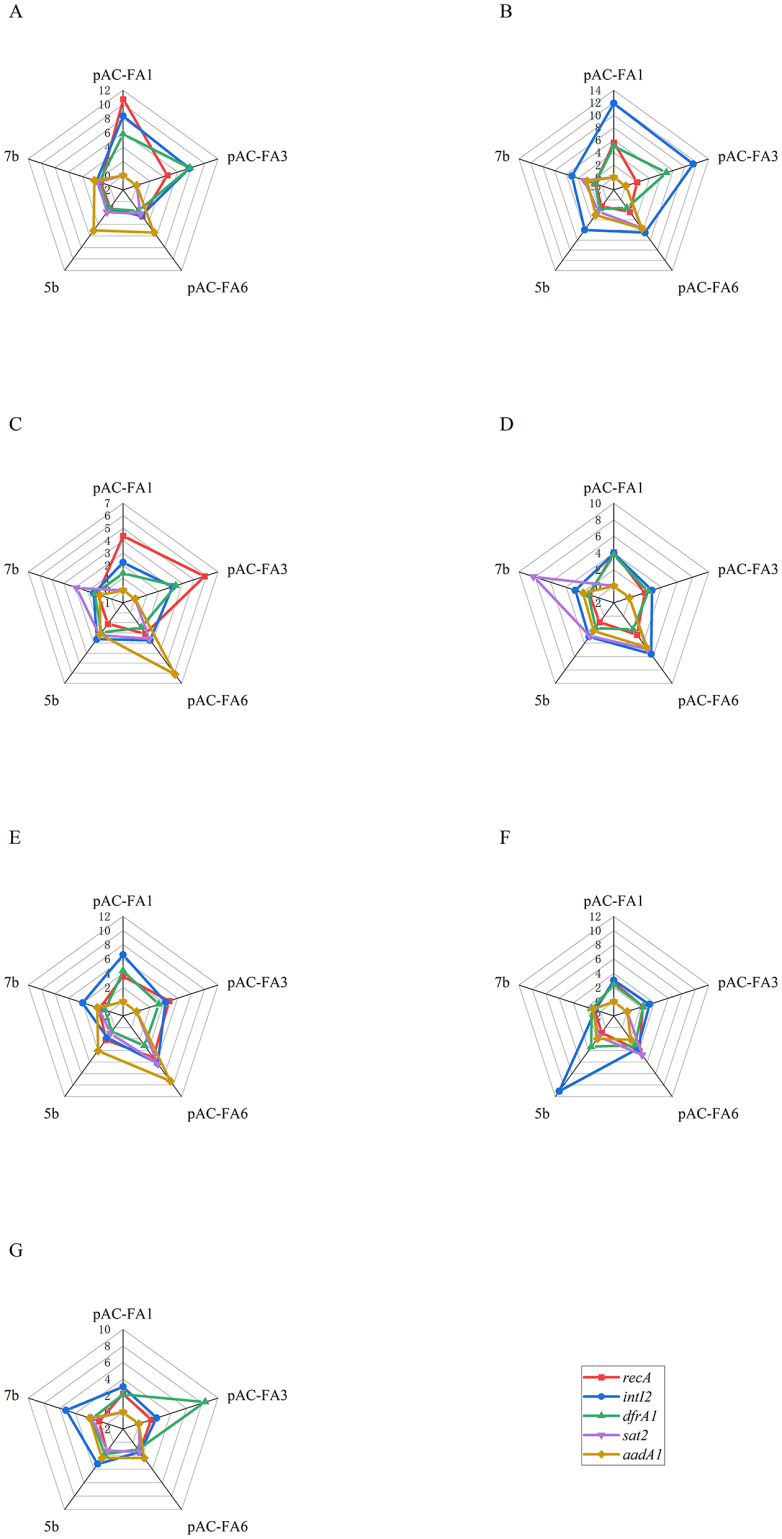
The relative expression of the target genes after 3 days of induction with different drugs. **(A)** 1/2 MIC of CIP. **(B)** 1/4 MIC of CIP. **(C)** 1/2 MIC of AMP. **(D)** 1/4 MIC of AMP. **(E)** 1/2 MIC of KAN. **(F)** 1/4 MIC of KAN. **(G)** MMC. Constructed strains pAC-FA1 and pAC-FA3 lack *sat2* and *aadA1* genes, which are represented as a value of 0.

After 1 day of culture induced by various drugs, the relative expression of *recA* in the experimental strains all increased to different extents (Figure 1). The constructed strain pAC-FA6 exhibited the most significant increases, with inductions by all drugs, except 1/4 MIC of AMP, reaching 5–12 times the pre-induction levels (*P* < 0.05), with KAN and MMC having the strongest effects. Constructed strains pAC-FA1 and pAC-FA3, which are structurally similar, exhibited similar induction patterns. The relative expression after induction by all drugs, except MMC, was 2–5 times higher than pre-induction levels. CIP had the strongest induction effect on pAC-FA1, whereas the induction effects of the three drugs on pAC-FA3 were not significantly different. In the clinical isolates 5b and 7b, MMC exhibited the most substantial effect on *recA* expression, reaching 4–6 times the pre-induction level (*P* < 0.001). In strain 5b, 1/4 MIC treatments did not result in a significant increase in the relative expression of *recA* after 1 day of induction, but continuous induction led to a sustained increase in expression. Compared to 1/4 MIC drug concentrations, 1/2 MIC concentrations more effectively increased *recA* expression, with significant differences observed in the induction capabilities among the different drugs (Figure 2). The trend generally showed a clear increase in *recA* expression on Day 1, peaking on Day 3, and slightly decreasing by Day 8.

### 3.3 Relative expression level of integrase and resistance genes

**Figure 3:**
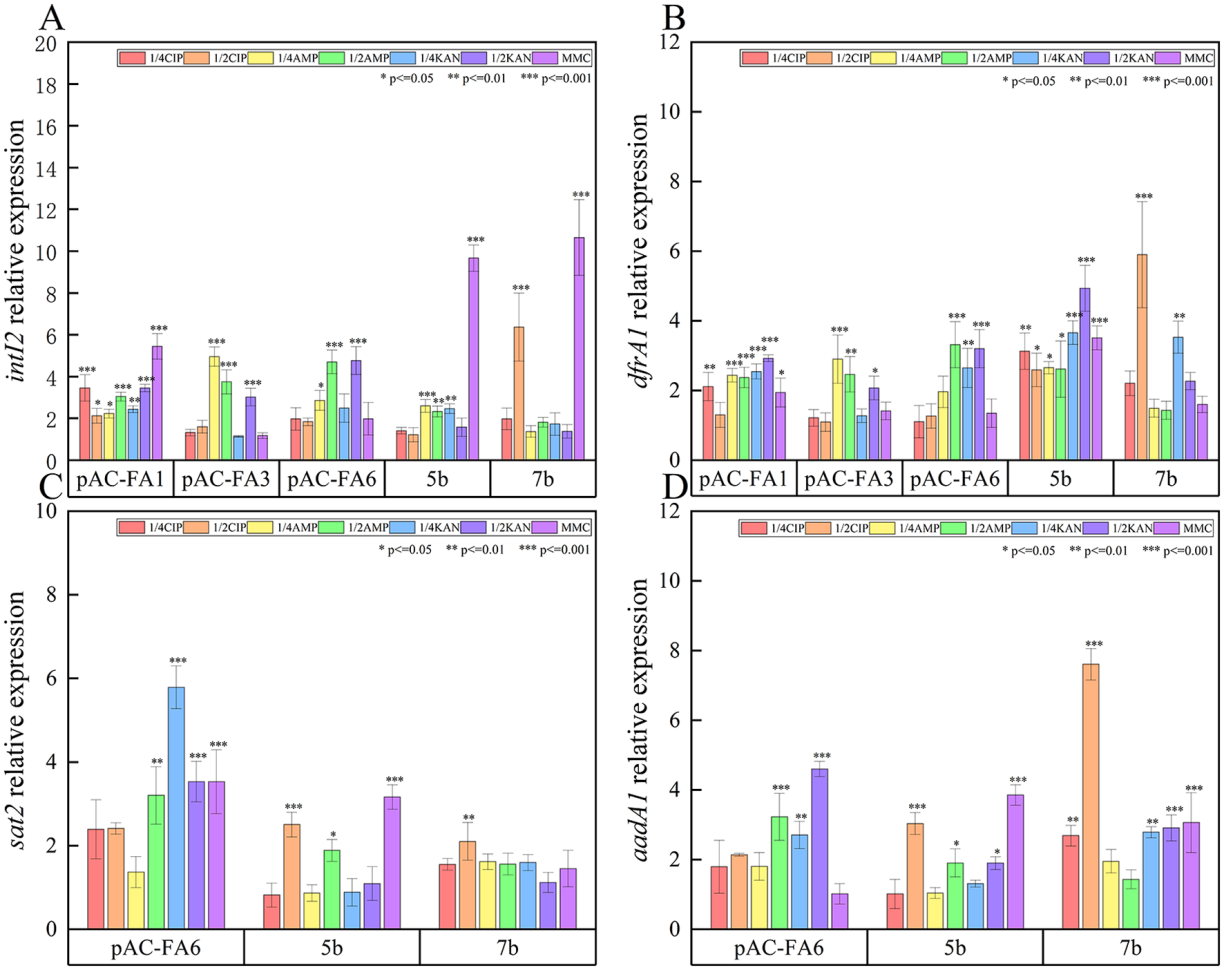
Relative expression of integrase (*intI2*) and resistance genes (*dfrA1, sat2* and *aadA1*). **(A)** The relative expression level of *intI2* after different drug-induced cultivation of experimental strains 1 day. **(B)** The relative expression level of *dfrA1* after different drug-induced cultivation of experimental strains 1 day. **(C)** The relative expression level of *sat2* after different drug-induced cultivation of experimental strains 1 day. **(D)** The relative expression level of *aadA1* after different drug-induced cultivation of experimental strains 1 day. The vertical axis values represent the ratio of relative expression of target genes in the experimental group to the control group. ***P < 0.001, **P < 0.01, *P < 0.05.

*intI2* (Figure 3A): In pAC-FA1, the relative expression level of *intI2* increased to 2–5 times the pre-induction level after drug-induced culture, with statistically significant differences observed (*P* < 0.05). In contrast, in pAC-FA3 and pAC-FA6, a statistically significant increase in *intI2* expression was only noted after induction with AMP and 1/2 MIC KAN (*P* < 0.001). In strains 5b and 7b, MMC was the most potent inducer of *intI2* expression, achieving levels more than 10 times the pre-induction levels. Furthermore, the increase in *intI2* expression was more pronounced than that of the resistance genes following the same drug induction (Figure 2).

*dfrA1* (Figure 3B): In the constructed strains, the pattern of relative expression changes in *dfrA1* after induction with various drugs mirrored that of *intI2*. In strain 5b, all drugs significantly induced an increase in *dfrA1* expression, reaching levels 2-5 times higher than those before induction (*P* < 0.05). In strain 7b, only 1/2 MIC of CIP and 1/4 MIC of KAN led to significant increases in *dfrA1* expression, while other conditions did not result in notable changes.

*sat2* (Figure 3C): In pAC-FA6, the relative expression level of *sat2* increased to 2–6 times the pre-induction level following induction by all drugs except for 1/4 MIC of AMP, with KAN having the most pronounced effect. In strain 5b, the relative expression level of *sat2* increased to 2–3 times the pre-induction level only after induction with 1/2 MIC concentrations of CIP, AMP, and MMC, with statistically significant differences (*P* < 0.05). In strain 7b, the increase in the relative expression level of *sat2* was not substantial.

*aadA1* (Figure 3D): In pAC-FA6, KAN, and AMP at 1/4 MIC led to a significant 3–5 fold increase in *aadA1* expression compared to pre-induction levels (*P* < 0.01). In strain 5b, MMC, along with 1/2 MIC concentrations of CIP, AMP, and KAN, caused a substantial rise in *aadA1* expression (*P* < 0.05). In strain 7b, induction with all agents except AMP led to a significant increase in *aadA1* expression, with CIP at 1/2 MIC inducing more than a 7-fold increase post-induction (*P* < 0.01).

### 3.4 Trends in changes of integrase and resistance genes

**Figure 4:**
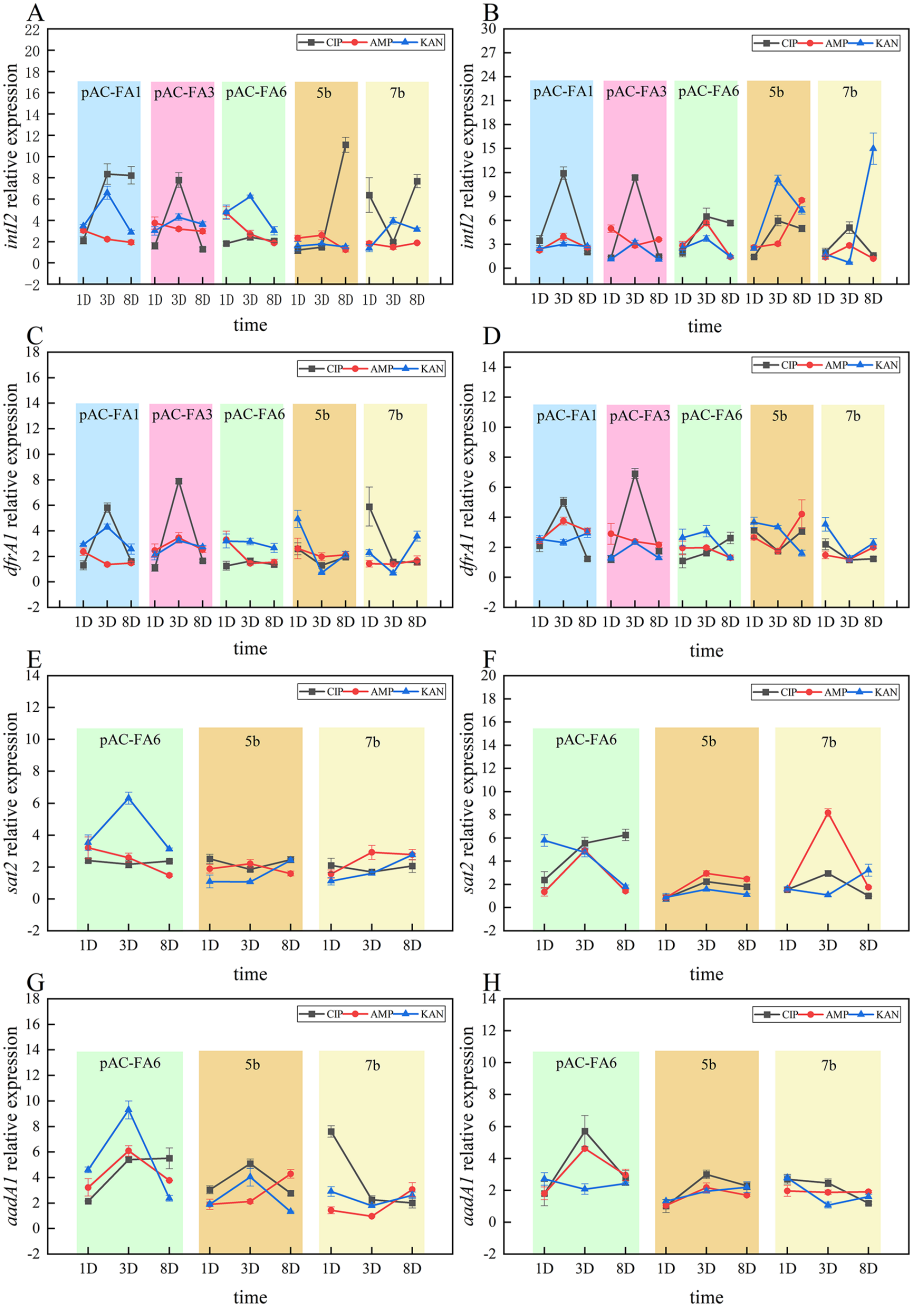
Trends in changes of integrase (*intI2*) and resistance genes (*dfrA1, sat2* and *aadA1*). **(A, C, E, G)** illustrate the changes in relative expression levels of the *intI2, dfrA1, sat2*, and *aadA1* genes, respectively, in the experimental strain under a 1/2 MIC concentration on days 1, 3, and 8. **(B, D, F, H)** show the changes in relative expression levels of the *intI2, dfrA1, sat2*, and *aadA1* genes, respectively, under a 1/4 MIC concentration on the same days. The vertical axis values represent the ratio of the relative expression of the target genes in the experimental group to the control group. The *p*-values and effect sizes are listed in Supplementary Table S3.

*intI2* (Figures 4A, B): In the constructed strains, drug-induced *intI2* expression generally increased initially, peaked on day 3, and then declined. In most instances, CIP demonstrated the strongest induction. For clinical isolates, CIP was the most potent inducer at 1/2 MIC, while KAN was most effective at 1/4 MIC.

*dfrA1* (Figures 4C, D): In constructed strains, *dfrA1* expression followed a similar pattern, with an initial increase, peaking on day 3, and then decreasing. In strains 5b and 7b, the most pronounced increase in relative expression occurred on day 1, with the induction effects of all three drugs being relatively similar.

*sat2* (Figures 4E, F): Changes in the relative expression of *sat2* following drug induction were not significant. In pAC-FA6, at a 1/2 MIC concentration, KAN demonstrated stronger induction than CIP and AMP, whereas, at a 1/4 MIC concentration, the induction effects of the three drugs were more similar. In strains 5b and 7b, the induction effects of the three drugs were relatively comparable.

*aadA1* (Figures 4G, H): In pAC-FA6, *aadA1* expression showed the most significant changes following KAN induction at 1/2 MIC, peaking on day 3. At 1/4 MIC, there was no significant difference in the effects of the three drugs, but all showed a marked increase on day 3. In clinical isolates, no significant increase in *aadA1* expression was observed, except for strain 7b induced by CIP at 1/2 MIC, and no consistent trend in changes was noted.

### 3.5 Changes in antibiotic resistance after induction with different drugs

**Figure 5:**
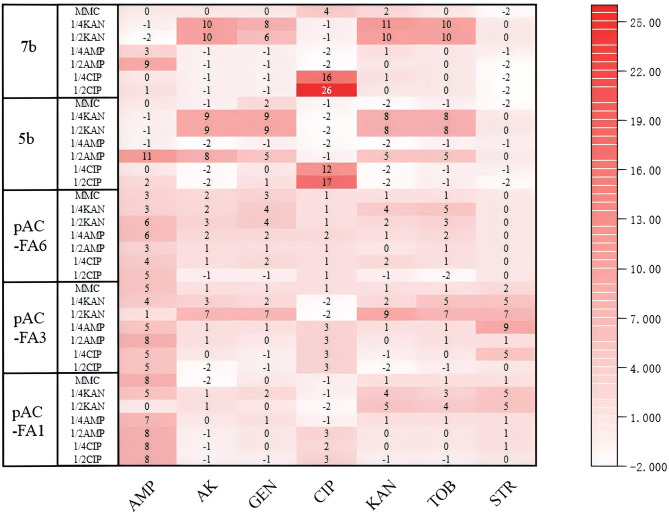
Changes in inhibition zones of experimental strains. The values in the figure represent the differences in inhibition zone sizes of the experimental strains before and after 8 days of drug induction. The vertical axis denotes the experimental strains and the inducing drugs, while the horizontal axis indicates the drugs measured.

In pAC-FA1, induction with CIP, AMP, and MMC resulted in a shift from sensitivity to resistance to AMP, while changes to other drugs were not significant. KAN induction caused pAC-FA1 to become resistant to certain aminoglycosides. In pAC-FA3 and pAC-FA6, the trends before and after induction were similar to those observed in pAC-FA1. In strains 5b and 7b, CIP induction led to a transition from sensitivity to resistance to CIP, especially at a 1/2 MIC concentration, where the effect was more pronounced. When induced with 1/2 MIC of AMP, strains 5b and 7b shifted from sensitivity to intermediate resistance to AMP, whereas induction with 1/4 MIC did not alter AMP resistance. After KAN induction, 5b and 7b transitioned from intermediate to resistant to some aminoglycosides (Figure 5).

### 3.6 Structural changes in integrase after induction with different drugs

**Figure 6:**
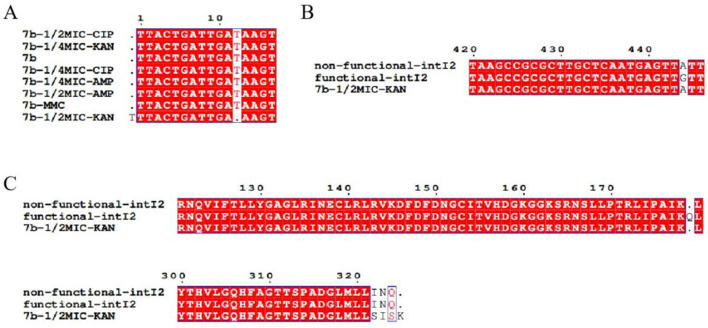
Sequencing results of integrase. **(A)** Post-induction with 1/2 MIC of KAN, the integrase of strain 7b exhibits a deletion of one base compared to its pre-induction sequence. **(B)** A mutation from A to G is observed at the 444 bp position in the sequence of the functional class 2 integrase. **(C)** Differences in the translated amino acid sequences of 7b and functional class 2 integrons before and after induction with 1/2 MIC of KAN.

Alignment of integrase sequences in 7b, conducted before and after drug induction, revealed a missing base at position 12 bp in the integrase sequence following KAN induction at 1/2 MIC. During this induction experiment, no mutations were detected at position 444 bp of the integrase gene in either pAC-FA1 or 7b (Figure 6).

## 4 Discussion

Bacteria develop resistance under the pressure of antibiotics. The emergence and widespread clinical dissemination of resistant strains have become a significant challenge to public health (Wistrand-Yuen et al., 2018). Class 2 integrons typically harbor three resistance gene cassettes: *dfrA1, sat2*, and *aadA1*, which confer resistance to trimethoprim, streptothricin, and aminoglycosides, respectively (Jové et al., 2017). The *intI2* integrase of non-functional class 2 integrons contains a stop codon, TAA, inserted after the 178th amino acid, rendering this class of integrases incapable of integrating and excising resistance gene cassettes, resulting in a truncated integrase protein. In contrast, functional class 2 integrons possess a stop codon mutation from TAA to CAA in the integrase gene, enabling the production of an active integrase, which facilitates the acquisition or rearrangement of gene cassettes.

Bacteria exposed to antibiotics can induce the SOS response (Sheng et al., 2021; Van Alstine and Sandler, 2022), which serves to maintain genomic integrity and enhance survival chances (Blázquez et al., 2018). Numerous studies on class 1 integrons have shown that fluoroquinolones, trimethoprim, and β-lactams can trigger the SOS response, activating horizontal gene transfer elements, including integrons (Ahmadian et al., 2020), thereby facilitating the spread of antibiotic resistance genes (Phillips et al., 1987; Goerke et al., 2006; Maiques et al., 2006; Mohanraj and Mandal, 2022). Drug concentrations below the minimum inhibitory concentration (MIC) are referred to as sub-inhibitory concentrations (sub-MIC) (Davies et al., 2006; Gu et al., 2020). The widespread clinical use of antibiotics (Patel et al., 2019; Singh and Bhunia, 2019; Polianciuc et al., 2020) as well as their improper use in agriculture and animal husbandry (Shen et al., 2008; Liu et al., 2023), results in low-level antibiotic exposures (sub-MIC) that are insufficient to kill bacteria but can induce changes in gene expression, leading to phenotypic changes (Sato et al., 2018; Davarzani et al., 2021) and increased mutation rates (Drlica, 2003; Drlica and Zhao, 2007; Gullberg et al., 2011; Barrett et al., 2019), thereby enhancing the potential for adaptive genetic variations. Studies have indicated that sub-MIC levels of antimicrobial agents may be more significant in contributing to bacterial resistance than lethal concentrations (Andersson and Hughes, 2012; Hughes and Andersson, 2012; Laureti et al., 2013). Class 2 integrons, as key mobile elements carrying resistance gene cassettes, are critically linked to the dissemination of resistance genes. This study systematically explores the dynamic relationship between SOS stress response, integron expression, and resistance phenotypes by inducing constructed strains (pAC-FA1, pAC-FA3, pAC-FA6) and clinical isolates (5b, 7b) with functional or non-functional class 2 integrons using two sub-MIC concentrations of antibiotics and mutagens (AMP, CIP, KAN, MMC). The findings provide experimental evidence for understanding the mechanisms of resistance evolution under low-level antibiotic exposure *in vitro*.

The SOS response plays a critical role in protecting bacterial communities from extreme environmental conditions, mediating survival and adaptation to genotoxic stress (Miura et al., 2020; Niccum et al., 2020), and contributing to the development of antibiotic resistance (Maslowska et al., 2019; Podlesek and Žgur Bertok, 2020; Xu et al., 2024). This response is a widespread regulatory network controlled by various regulatory proteins and enzymes and is highly conserved across different pathogens. Among these, RecA and LexA are essential proteins required universally for DNA damage-mediated SOS repair in bacteria. Even under non-inducing conditions, RecA is expressed at basal levels, but in induced cells, RecA rapidly binds to ssDNA and facilitates the degradation of LexA, ensuring a swift and effective SOS response (Ghodke et al., 2019). The expression level of *recA* is a key indicator of SOS response activation. In this study, the SOS response was monitored by measuring the relative expression levels of *recA* using real-time quantitative PCR. Our experiments revealed that the relative expression of *recA* in experimental strains increased to varying degrees following induction with various drugs, exhibiting both concentration and time dependency. Drug concentrations at 1/2 MIC were more effective in elevating *recA* expression than 1/4 MIC concentrations. Comparing days 1, 3, and 8 of induction showed that *recA* expression significantly increased after just 1 day, peaked on the third day, and slightly decreased by the eighth day. This dynamic change suggests that bacteria can quickly mobilize related genes to trigger the SOS response when faced with adverse conditions, aiding in their survival until they adapt to the stressor. There were notable differences in how different strains responded to the drugs: after CIP induction, pAC-FA1 exhibited the greatest increase in *recA* expression. In pAC-FA3, the effects of the drugs were relatively similar, while in pAC-FA6, KAN had the most notable effect. In strains 5b and 7b, MMC showed the strongest induction effect. These findings demonstrate that all four antibiotics tested in this experiment can enhance *recA* expression, thereby triggering the bacterial SOS response to combat adverse conditions. However, the tolerance of each strain to different drugs varies, and even at the same concentration, different levels of SOS response may be triggered to counteract these stressors. Additionally, although the relative expression of *recA* was highest in pAC-FA6, the change in resistance after 8 days of continuous antibiotic induction was not the most significant. The high heterogeneity within bacterial populations (Orman and Brynildsen, 2013), coupled with potential indirect feedback inhibition and differences in regulatory proteins due to different antibiotic mechanisms (Schaenzer and Wright, 2020; Wilson et al., 2020; Huang et al., 2022; Baranova et al., 2023), may collectively contribute to a non-linear relationship between *recA* levels and resistance changes, underscoring the need for further research in this area. Under identical induction conditions, differences in the relative expression levels of *recA* were observed between the constructed strains and clinical isolates, with the constructed strains showing a more pronounced increase. The survival mechanisms of cellular stress responses are closely linked to bacterial metabolism (Recacha et al., 2024). We hypothesize that *E. coli* TOP10, being a genetically engineered strain, has well-developed regulatory systems such as cAMP-CRP (Heyde et al., 2021) and LexA (Cory et al., 2024). Engineered expression vectors often contain strong promoters and terminators, and the strain's faster growth rate can support the higher energy demands of foreign protein expression. In contrast, the natural genome of *Proteus mirabilis* may have target genes regulated by upstream inhibitory elements, leading to slower growth and a greater allocation of energy to basic metabolism rather than foreign gene expression.

After drug induction, the relative expression levels of class 2 integron integrase and resistance genes in the variable region of experimental strains increased to varying degrees. In the constructed strains pAC-FA1 and pAC-FA3, CIP exhibited the strongest induction effect compared to AMP and KAN, generally showing an initial increase with a peak on day 3, followed by a decline. In pAC-FA6, the induction effects of all four drugs were relatively similar, although KAN specifically led to a noticeable rise in the expression levels of *sat2* and *aadA1*. In clinical isolates, 5b and 7b, MMC strongly induced expression of the class 2 integron integrase gene, whereas the other three drugs did not demonstrate significant differences in their effects. It can be observed that non-functional *intI2*, although unable to modify its gene cassettes array, can still effectively express the gene cassettes (Jové et al., 2017). The expression of integrases must be regulated, “turning on” and “off” as required to obtain the functional diversity encoded by gene cassettes. From an evolutionary perspective, the ability of *intI2* to upregulate transcription in strains with non-functional integrases may serve as a stress reserve (Escudero et al., 2015). Moreover, even among constructed strains, the same drug can exert substantially different effects. It is hypothesized that the presence of non-functional class 2 integrons, functional class 2 integrons, and even the number of resistance genes linked to class 2 integrons might influence the adaptive capabilities of bacteria, leading to variations in growth rates and drug sensitivities. As independent clinical isolates, 5b and 7b exhibit inherent genomic differences beyond the presence of class 2 integrons. The genetic heterogeneity of clinical strains may also lead to differential responses in stress regulatory networks, such as the SOS response system, to the same antibiotic concentration, resulting in inconsistent upregulation trends of resistance genes between the two strains. Further experiments are needed to confirm these hypotheses.

Integrons facilitate the rapid dissemination of bacterial resistance by capturing and expressing resistance gene cassettes, and their activity is regulated by environmental stress. This study found that, following drug-induced culture, the relative expression levels of class 2 integron integrase and resistance genes in the variable region of experimental strains increased to varying degrees, aligning with the expression trend of *recA* (Figures 2–4). This suggests a potential association between the expression of class 2 integrons and the SOS response. Research on class 1 integrons has identified a potential LexA binding site on the *intI1* promoter, suggesting that the SOS response can specifically and directly induce the expression of class 1 integrase (Guérin et al., 2010). In class 2 integrons, a potential LexA binding site overlaps with the P*intI2* promoter; however, subsequent experiments have shown that the LexA protein does not bind to this region. The antibiotic-induced SOS response could activate other LexA binding sites within the bacterial genome, such as promoters of DNA repair genes, thereby indirectly promoting the expression of *intI2* and resistance genes through shared regulatory networks like RecA-mediated transcription factor activation. The expression of integrons is closely linked to bacterial physiology and environmental conditions, adapting as needed. Additionally, class 2 integrons might possess alternative stress-sensing mechanisms, potentially regulating expression by responding to other stress signaling pathways like oxidative stress or membrane damage response. This hypothesis requires validation through future transcriptomic or protein interaction studies (Erill et al., 2007).

The inhibition zone assay demonstrated that sub-MIC concentrations of antibiotics can significantly alter bacterial resistance phenotypes (Figure 5). After AMP induction, the sensitivity of constructed strains pAC-FA1 and pAC-FA3 to AMP decreased markedly, shifting from sensitive to resistant/intermediate. Clinical isolates 5b and 7b exhibited significantly enhanced resistance to CIP, with a reduction in the inhibition zone size. This finding supports the conclusion that low-level antibiotic exposure can drive resistance evolution (Barrett et al., 2019; Anderson et al., 2023). The induction effect at 1/2 MIC concentration was stronger than at 1/4 MIC, suggesting that higher sub-MIC concentrations exert greater selective pressure on resistance genes, thereby more readily promoting the emergence of resistant phenotypes. The differences in resistance between clinical and constructed strains are also noteworthy. Clinical isolates rapidly acquired resistance to the corresponding drugs after induction (especially with CIP), while the phenotypic changes in constructed strains were more limited. This may be attributed to the accumulation of gene elements, such as multiple mobile genetic elements, in clinical strains through long-term natural evolution, allowing for more efficient mobilization of resistance genes in response to stress. In contrast, constructed strains, with simpler genomes like that of *E. coli* TOP10, carrying only a single integron element, have limited adaptive evolutionary capacity. Additionally, some strains exhibited increased resistance to unrelated antibiotics after induction with a specific drug, corroborating the idea that antibiotics can promote multidrug resistance through “cross-selection” (Blázquez et al., 2018).

Sequencing of the class 2 integrase genes before and after induction did not reveal mutations in the stop codon TAA, which is inserted after the 178th amino acid in the integrase gene. The evolutionary capacity of bacteria depends not only on the rate at which genetic diversity is generated but also on the population size (Blázquez et al., 2018). In clinical and environmental settings, bacteria are often continuously exposed to multiple antibiotics, with pressure intensity dynamically changing over time. This “chronic mixed pressure” can accelerate the accumulation of adaptive mutations (Good et al., 2017; Lenski, 2017). Short-term antibiotic exposure may not lead to mutations at critical sites of class 2 integrons. Additionally, it is possible that any induced mutations are of low frequency and were not detected by the methods employed in this study. Occasionally, tester strains exhibited a base deletion downstream of the stop codon; however, due to the presence of the stop codon, their integrase gene would not have translated an intact integrase protein, rendering the mutation apparently ineffective on the integrase function. Further investigation is required to elucidate the mutation mechanisms of functional class 2 integrons.

## 5 Conclusion

Sub-MIC concentrations of drugs have been shown to induce an increase in the relative expression of *recA*, associated with the bacterial SOS response. This induction also elevates the expression levels of class 2 integron integrase and resistance genes. Continuous induction results in a sustained upregulation of these genes, which stabilizes or slightly decreases upon reaching a plateau, ultimately driving the evolution of clinical-level resistance in different antibiotic classes with various cellular targets. Moreover, higher sub-MIC levels more effectively trigger bacterial responses to adverse environments, enhancing population survival. The induction capacities of different drugs vary significantly, and the underlying mechanisms require further investigation. Short-term exposure to a single antibiotic did not result in critical mutations that would transform class 2 integrons into a functional form. The widespread use of antimicrobial agents in clinical settings, agriculture, and livestock inevitably leads to low-level antibiotic exposure, providing sufficient evolutionary pressure for bacterial resistance. It is imperative to develop effective, targeted strategies to address this challenge.

## Data Availability

All key information data generated or analyzed during this study are included in this article/Supplementary material.
