# Supplementary material for: The expression regulation of recA gene and bacterial class 2 integron-associated genes induced by antibiotics
Source: Front Microbiol. 2025 Aug 29;16:1632813. doi: 10.3389/fmicb.2025.1632813 (PMC12425958; doi:10.3389/fmicb.2025.1632813)
Supplement: Supplementary file 1 [file Table_1.docx]

**Supplementary Table S1**: Oligonucleotide primers used in this study

| Primer | Sequence （5′→3′） | target gene | Length(bp) |
| --- | --- | --- | --- |
| intIF2 | TTACTGATTGATAAGTAGCATCAGTCCACT | class 2  integron | 5170 |
| intIB2 | TCGAGAGAGGATATGGAAGG |  |  |
| intl2F | CGGATGATGACTCTGATGAAGACTCTG | intI2 attC1 | 1877 |
| FA1-R | TTTGTCACCCGCTGATGCTTACC |  |  |
| FA6-R | CGGTGTCGGCTTGAATGAATTGTTAG | intI2 attC3 | 3323 |
| recA-F | GATGAAGATCAGCAGCGTGTTGG | recA | - |
| recA-R | AAGGCGAAATCGGCGACTCTC |  |  |
| intI2-F | GCGTGGTCTTAACATCGGTATGC | intI2 |  |
| intI2-R | CATCGTTAGCAAGCGTGTCAC |  |  |
| sat2-F | TCGGCGGCATTGACCTGTTC | sat2 |  |
| sat2-R | GTCATCCTGTGCTCCCGAGAAC |  |  |
| dfrA1-F | GGAGCATTACCCAACCGAAAGTATG | dfrA1 |  |
| dfrA1-R | CCCACCACCTGAAACAATGACATG |  |  |
| aadA1-F | CGACATCATTCCGTGGCGTTATC | aadA1 |  |
| aadA1-R | CGTGGCTGGCTCGAAGATACC |  |  |
| 16SrRNA-F | TGGGGAGCAAACAGGATTAGATACC | 16SrRNA |  |
| 16SrRNA-R | CGGAAGCCACGCCTCAAGG |  |  |
| cat-F | GGAGTGAATACCACGACG | cat |  |
| cat-R | GGATTGGCTGAGACGAA |  |  |

**Supplementary Table S2**: The initial inhibition zone of the experimental strains

| experimental strain | AMP | AK | GEN | CIP | KAN | TOB | STR | TM |
| --- | --- | --- | --- | --- | --- | --- | --- | --- |
| pAC-FA1 | 20 | 21 | 20 | 35 | 20 | 19 | 15 | 6 |
| pAC-FA3 | 19 | 22 | 20 | 36 | 20 | 19 | 15 | 6 |
| pAC-FA6 | 20 | 23 | 21 | 37 | 20 | 19 | 6 | 6 |
| 5b | 26 | 17 | 17 | 32 | 15 | 15 | 6 | 6 |
| 7b | 26 | 18 | 16 | 32 | 18 | 16 | 6 | 6 |
